# Genomics of Ocular *Chlamydia trachomatis* After 5 Years of SAFE Interventions for Trachoma in Amhara, Ethiopia

**DOI:** 10.1093/infdis/jiaa615

**Published:** 2020-10-09

**Authors:** Harry Pickering, Ambahun Chernet, Eshetu Sata, Mulat Zerihun, Charlotte A Williams, Judith Breuer, Andrew W Nute, Mahteme Haile, Taye Zeru, Zerihun Tadesse, Robin L Bailey, E Kelly Callahan, Martin J Holland, Scott D Nash

**Affiliations:** 1 Department of Clinical Research, London School of Hygiene and Tropical Medicine, London, United Kingdom; 2 The Carter Center, Addis Ababa, Ethiopia; 3 Division of Infection and Immunity, University College London, London, United Kingdom; 4 The Carter Center, Atlanta, Georgia, USA; 5 Amhara Public Health Institute, Bahir Dar, Ethiopia

**Keywords:** trachoma, *Chlamydia trachomatis*, antimicrobial resistance, genomics, whole-genome sequencing, mass drug administration, azithromycin, neglected tropical disease

## Abstract

**Background:**

To eliminate trachoma as a public health problem, the World Health Organization recommends the SAFE (surgery, antibiotics, facial cleanliness, and environmental improvement) strategy. As part of the SAFE strategy in the Amhara Region, Ethiopia, the Trachoma Control Program distributed >124 million doses of antibiotics between 2007 and 2015. Despite this, trachoma remained hyperendemic in many districts and a considerable level of *Chlamydia trachomatis* (*Ct*) infection was evident.

**Methods:**

We utilized residual material from Abbott m2000 *Ct* diagnostic tests to sequence 99 ocular *Ct* samples from Amhara and investigated the role of *Ct* genomic variation in continued transmission of *Ct*.

**Results:**

Sequences were typical of ocular *Ct* at the whole-genome level and in tissue tropism–associated genes. There was no evidence of macrolide resistance in this population. Polymorphism around the *ompA* gene was associated with village-level trachomatous inflammation–follicular prevalence. Greater *ompA* diversity at the district level was associated with increased *Ct* infection prevalence.

**Conclusions:**

We found no evidence for *Ct* genomic variation contributing to continued transmission of *Ct* after treatment, adding to evidence that azithromycin does not drive acquisition of macrolide resistance in *Ct*. Increased *Ct* infection in areas with more *ompA* variants requires longitudinal investigation to understand what impact this may have on treatment success and host immunity.

## Teaser

Whole-genome sequencing of ocular *Chlamydia trachomatis* from Amhara, Ethiopia, found no evidence of macrolide resistance, despite 5 years of trachoma control interventions, including mass distribution of azithromycin. Polymorphisms in *ompA* were associated with prevalence of ocular infection and trachomatous disease.

Trachoma is a blinding disease caused by *Chlamydia trachomatis* (*Ct*). To eliminate trachoma as a public health problem, the World Health Organization (WHO) recommends the SAFE (surgery, antibiotics, facial cleanliness, and environmental improvement) strategy [1]. This includes annual mass drug administration (MDA) of azithromycin to individuals aged ≥6 months and topical tetracycline eye ointment for pregnant women and children aged <6 months. The number of recommended years of interventions is based on prevalence of trachoma in a district [2]. For districts considered hyperendemic for trachoma, defined as a trachomatous inflammation–follicular (TF) prevalence of ≥30% among children aged 1–9 years, 5–7 years of SAFE are recommended followed by further population-based surveys to determine the impact of the interventions.

As part of the SAFE strategy in Amhara National Regional State, Ethiopia, the Trachoma Control Program distributed >124 million doses of antibiotics from 2007 to 2015 [3]. Both administrative and self-reported coverage have demonstrated treatment coverage close to or above the WHO-recommended threshold of 80% [3–5]. The program also provided health education and assisted in the construction of latrines as part of the F and E components of SAFE [3]. Despite an average of 5 years of these interventions, trachoma remained hyperendemic in many districts, with considerable levels of *Ct* infection [6].

Historically, *Ct* molecular epidemiology focused on *ompA* [7], which encodes the major outer membrane protein. More recently, multilocus sequence typing schemes have been used [8, 9]. Since 2010, there has been a rapid expansion of *Ct* whole-genome sequencing (WGS), due to the ability to sequence directly from clinical samples [10, 11]. Despite >700 *Ct* genomes being sequenced [12–15], few studies have evaluated the role of genome-level variation in *Ct* transmission and outcomes of infection. Recent publications have begun to address these questions in *Ct* from trachoma-endemic settings [15, 16]. WGS additionally allows monitoring of antimicrobial resistance in *Ct* [16–18], which is of critical importance as MDA with azithromycin is key for trachoma control and is under consideration as an intervention for childhood mortality [19], neonatal sepsis [20], and malaria [21].

The Trachoma Control Program in Amhara has conducted multiple studies to better understand the epidemiology of trachoma in communities that have received approximately 5 years of annual MDA, yet still have significant levels of *Ct* infection and disease. This study sequenced 99 ocular *Ct* samples from Amhara to identify antimicrobial resistance alleles and investigate the role of genomic variation in the continued transmission of *Ct*. We further explored the relationship between *Ct* genomic variation, ocular *Ct* infection prevalence, and trachomatous disease prevalence at the village and district levels.

## MATERIALS AND METHODS

### Study Design and Population

Between 2007 and 2010, the SAFE strategy was scaled to reach all Amharan districts with interventions administered for 5 years. Methodology for these district-level surveys has been published previously [3]. In brief, a multistage cluster randomized methodology was used, whereby clusters (villages) were selected using a population proportional to estimated size method, and within a cluster, a segmentation approach was used to randomly select 30–40 households [3].

After enumerating all residents, consented residents were examined for trachoma. Every other cluster was chosen for swab collection prior to surveying a district, and during the house-to-house survey, the first 25 children aged 1–5 years with parental consent were swabbed for the presence of infection. If >1 child aged 1–5 years lived in a household, 1 child was randomly chosen by survey software.

### Sample Collection and Processing

Gloved graders swabbed the upper tarsal conjunctiva 3 times with a polyester-tipped swab, rotating 120 degrees along the swab’s axis each time to collect a sufficient epithelial specimen [6]. Samples were transferred to the Amhara Public Health Institute (APHI) and stored at –20°C. Conjunctival swabs from each district were randomized and 5 samples were combined into each pool. Pools were processed with the real-time polymerase chain reaction (PCR) assay on the Abbott m2000 system (hereafter “RealTime Assay”) at the APHI laboratory [6]. All individual samples from positive pools from North Gondar, South Gondar, East Gojam, and Waghemra were processed again to provide individual-level data [22]. Samples from these zones were prioritized owing to the persistently high trachoma prevalence. For positive individual samples, the PCR cycle threshold was converted to *Ct* elementary body equivalent concentration based on a calibration curve of known elementary body concentrations on the RealTime Assay [22].

Once *Ct* load was known for the positive individual samples, a total of 240 with the highest load were chosen for this project. Samples with sufficient *Ct* load, likely to obtain high-quality full genome sequence data based on our previous studies, were reextracted as described below [15, 16, 23].

### *Ct* Detection and Sequencing Preparation

DNA was extracted from 800 µL residual material per sample from Abbott m2000 diagnostic tests using the QIAamp mini DNA kit. Samples were quantified using a genome target by quantitative PCR [24]. Samples with ≥10 genome copies per µL of DNA were considered for WGS.

### Sequencing, Processing, and Analysis of *Ct*

Sequencing was performed as previously described [15], except we utilized the SureSelectXT Low Input kit. Processing and analysis of sequenced reads was performed as previously described [16]. In brief, raw reads were trimmed and filtered using Trimmomatic. Filtered reads were aligned to a reference genome (A/Har13) with Bowtie2, and variants were called with SAMtools/BCFtools. Multiple genome and plasmid alignments were generated using progressiveMauve, and multiple gene alignments were generated using MUSCLE. Phylogenies were computed using RAxML, and predicted regions of recombination were masked using Gubbins. Domain structure of *tarP* and truncation of *trpA* were characterized as previously described [15]. ABRicate and the ResFinder database were used to identify antimicrobial resistance genes in the reference-assembled genomes and de novo assembled reads.

### Genome-Wide Association Analyses

Genome-wide association analysis (GWAS) was performed to identify polymorphisms specific to this population of ocular *Ct* sequences through comparison of 99 Amharan *Ct* genomes to 213 previously sequenced samples from trachoma-endemic communities. Heterozygous calls and positions with >25% missing data were removed. Polymorphisms were considered conserved in Amhara if the major allele frequency was >0.8 and rare in the representative ocular population if the same allele was at a frequency <0.2. The final analysis included 116 single-nucleotide polymorphisms (SNPs). A logistic regression was performed with each Amhara-specific site as the independent variable and origin of the sequence as the dependent variable (reference level; representative and comparator level; Amharan). *P* values were Bonferroni corrected.

GWAS was performed to identify *Ct* polymorphisms associated with village-level clinical data. Heterozygous base calls and positions with a minor allele frequency of <25% or >25% missing data were removed. The final analysis included 681 SNPs. A linear regression was performed with each SNP as the independent variable and village-level *Ct* infection, TF, or trachomatous inflammation–intense (TI) prevalence as the dependent variable. District was included as a random effect and with adjustment for age and sex. *P* values were Bonferroni corrected. Additionally, a sliding-window approach was used to identify polymorphic regions of the genome. Windows of 10 kilobases were evaluated, with a step size of 1 kilobase. The final analysis included 907 polymorphic regions. A linear regression was performed with each polymorphic region collapsed into a pseudo-haplotype per sequence as the independent variable, including district as a random effect and adjusted for age and sex. This model was compared to a model including only the covariates and random effects by F test. *P* values were Bonferroni corrected.

### Inference of *ompA* Sequences

Complete sequences of *ompA* were obtained from WGS data using the reference-based assembly method described above with 1 change. Each sample was assembled against 4 reference sequences (A/Har-13, B/Jali-20, C/TW-3, and D/UW-3), and the assembly with the highest coverage was used for downstream analyses. Serovar of *ompA* was assigned using maximum *blastn* homology against all published *Ct* sequences. Genotypes of *ompA* were manually determined using SeaView. Diversity of *ompA* genotypes was calculated as Simpson D using *vegan* in R.

### Ethical Considerations

Survey methods were approved by the Emory University Institutional Review Board (IRB) (protocol 079-2006) as well as the Amhara Regional Health Bureau. Due to high illiteracy rate among the population, approval was obtained for oral consent/assent. Oral consent/assent was recorded electronically for all participants according to the principles of the Declaration of Helsinki. Respondents were allowed to terminate the examination at any point without explanation. Further permission for sample transfer and genomic sequencing of *Ct* was provided by the Emory University IRB and the Amhara Regional Health Bureau. Human DNA testing and genotyping were not conducted on these samples. Any unused biological material will be returned to Ethiopia or disposed as required.

## RESULTS

Ocular swabs previously confirmed as positive for *Ct* DNA were selected for this study (n = 240); samples with sufficiently high concentration of *Ct* DNA after reextraction were considered for WGS (n = 135). Of these, 99 were randomly selected for sequencing to match the complete dataset on age, sex, and zone of collection. The sequenced and complete samples were comparable (Table 1), except as expected with a higher median load of infection in sequenced samples.

**Table 1.** Demographic and Trachoma Characteristics of Complete and Sequenced Samples—Amhara, Ethiopia, 2011–2015

Characteristic	Complete Dataset	Sequenced Dataset
	(n = 240)	(n = 99)
Median age, y, range	3 (1–5)	3 (1–5)
Female sex, No. (%)	224 (52.6)	48 (48.5)
Zone, No. (%)		
East Gojam	100 (41.7)	43 (43.4)
North Gondar	22 (9.2)	12 (12.1)
South Gondar	69 (28.8)	26 (26.3)
Waghemra	49 (20.4)	18 (18.2)
Median cluster TF prevalence, % (range)	58.8 (13.5–90.7)	58.7 (15.3–90.7)
Median cluster TI prevalence, % (range)	14.9 (0.0–51.4)	15.4 (0.0–51.4)
Median cluster *Ct* prevalence, % (range)	28.0 (4.0–100.0)	24.0 (4.0–100.0)
Median load of infection (range)^a^	368.9 (27.29–2.49 × 10^6^)	1431.2 (213.67–1.26 × 10^6^)

Abbreviations: *Ct*, *Chlamydia trachomatis*; TF, trachomatous inflammation–follicular; TI, trachomatous inflammation–intense.

^a^Elementary bodies per swab.

**Figure 1.**
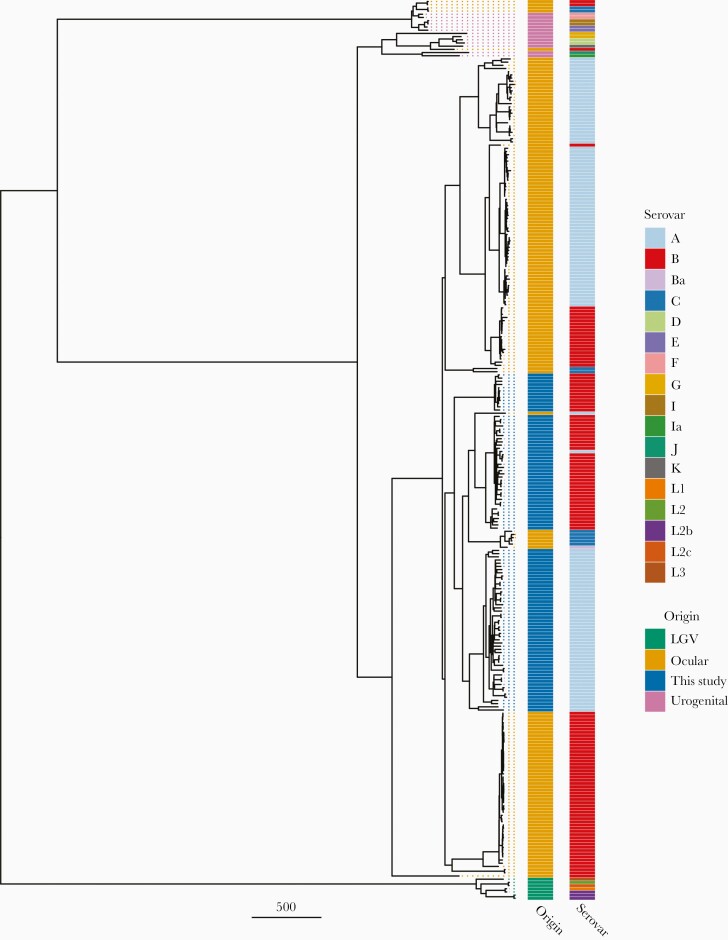
Maximum likelihood reconstruction of whole-genome phylogeny of ocular *Chlamydia trachomatis* (*Ct*) sequences from Amhara, Ethiopia. Whole-genome phylogeny of 99 *Ct* sequences from Amhara and 183 *Ct* clinical and reference strains. Amharan *Ct* sequences were mapped to *Ct* A/HAR-13 using Bowtie2. Single-nucleotide polymorphisms were called using SAMtools/BCFtools. Phylogenies were computed with RAxML from a variable sites alignment using a GTR + γ model and are midpoint rooted. The scale bar indicates pairwise distance. *Ct* sequences are colored by origin of the sample (“Origin”) and *ompA* serovar (“Serovar”). Abbreviation: LGV, ymphogranuloma venereum.

The Amharan *Ct* genomes formed 2 subclades within the T2 ocular clade (Figure 1). The 2 subclades were predominantly separated by *ompA* genotype, with 52 serovar A (SvA) and 47 serovar B (SvB) genomes. Focusing on genomes from ocular infections (Supplementary Figure 1), the SvA Amharan genomes branch together independent from any previously sequenced *Ct*. The SvB Amharan genomes were split across 2 branches. One branch was most closely related to A/Har-13, isolated from Saudi Arabia. The second, smaller branch was most closely related to Ba/Apache-2 from the United States as well as recently sequenced ocular *Ct* from the Solomon Islands.

Several *Ct* genes and genomic regions are hypothesized to be indicative of tissue tropism, with polymorphisms distinct to ocular, urogenital, and lymphogranuloma venereum (LGV) sequences. All Amharan *Ct* genomes had *tarP* domain structure typical of ocular sequences [25]. Similarly, all Amharan genomes had inactivating mutations in *trpA*, leading to a nonfunctional tryptophan synthase [26]. Polymorphic membrane proteins clustered phylogenetically with ocular isolates (Supplementary Figure 2) [27]. There was minimal polymorphism in the *Ct* plasmid within the Amharan genomes and they were closely related to previously sequenced ocular isolates (Supplementary Figure 3). There was no evidence for the presence of macrolide-resistance alleles in the assembled genomes or de novo assembled reads.

**Figure 2.**
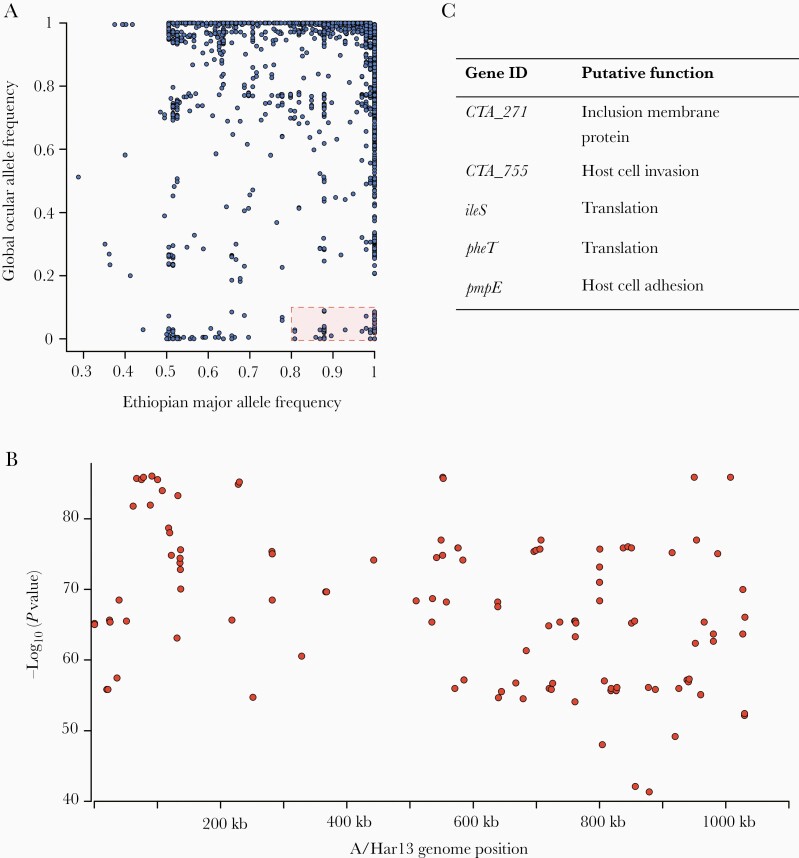
Single-nucleotide polymorphisms (SNPs) on the *Chlamydia trachomatis* (*Ct*) genome specific to Amhara, Ethiopia. *A*, SNPs conserved in Amhara (allele frequency ≥0.8) and rare in other *Ct* sequences (allele frequency ≤0.2) were identified by comparing these *Ct* sequences (n = 99) to ocular genomes from other populations (n = 213). *B*, Logistic regression found SNPs specific to this Amharan population to be dispersed throughout the genome (n = 116). *C*, Five genes harbored 3 Amhara-specific SNPs; putative function was determined by reference to published literature.

Amharan *Ct* genomes were compared to 213 previously sequenced samples from trachoma-endemic communities to identify genomic markers specific to Amhara [12–16, 23]. Of 36 805 polymorphic sites (Figure 2A), 116 were conserved in Amhara (frequency ≥0.8) and rare in the representative ocular population (frequency ≤0.2). These were dispersed throughout the genome (Figure 2B). Fourteen genes harbored 2 such sites and 5 genes contained 3 sites, all of which have previously been identified as polymorphic in distinct populations of *Ct* (Figure 2C).

A GWAS was performed to identify polymorphism within the Amharan *Ct* genomes related to village-level prevalence of *Ct* infection. The analysis included 681 SNPs in 99 genomes. No SNPs were associated with village-level prevalence of infection (Supplementary Figure 4). A secondary sliding-window approach was utilized to identify polymorphic regions of the genome associated with infection prevalence. The analysis included 907 polymorphic regions in 99 genomes. No polymorphic regions were associated with village-level prevalence of infection (Supplementary Figure 4).

**Figure 3.**
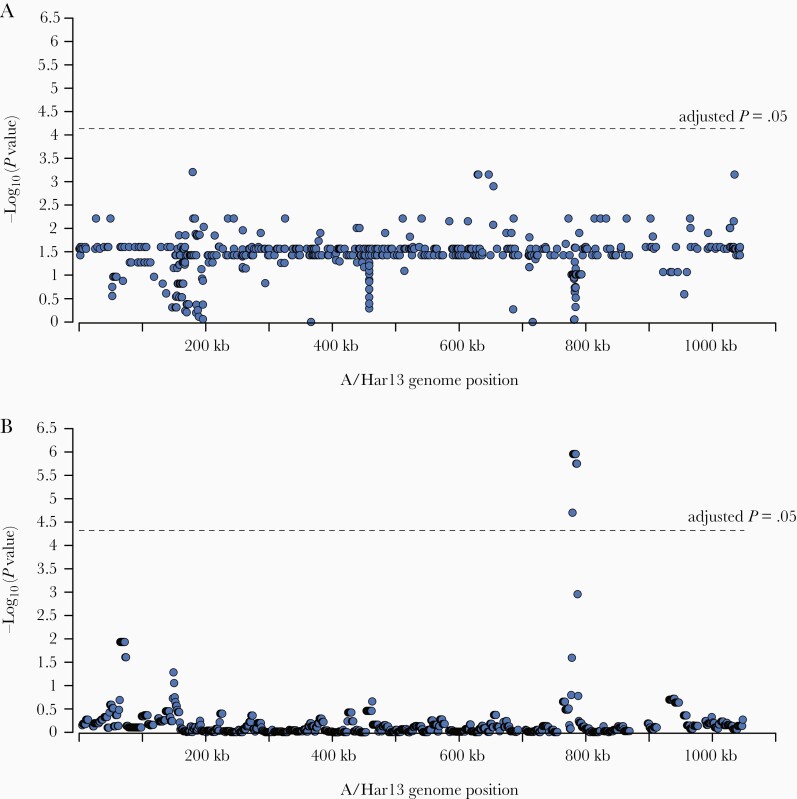
Polymorphisms on the *Chlamydia trachomatis* genome associated with village-level trachomatous inflammation–follicular (TF) prevalence. *A*, No single-nucleotide polymorphisms were significantly associated with village-level TF prevalence. *B*, Eight polymorphic regions from positions 774 000 to 791 000 were associated with village-level prevalence of TF.

No SNPs were associated with village-level prevalence of TF (Figure 3A). However, 8 polymorphic regions from positions 774 000–791 000 were associated with TF prevalence (Figure 3B). SNPs in these regions were focused in CTA0743/*pbpB* (harboring 29 SNPs), CTA0747/*sufD* (10 SNPs), and CTA0742/*ompA* (7 SNPs). All SNPs in *sufD* were synonymous, while 8 of 29 and 3 of 7 SNPs in *pbpB* and *ompA*, respectively, were nonsynonymous.

No SNPs or polymorphic regions were associated with village-level prevalence of TI (Supplementary Figure 5).

**Figure 4.**
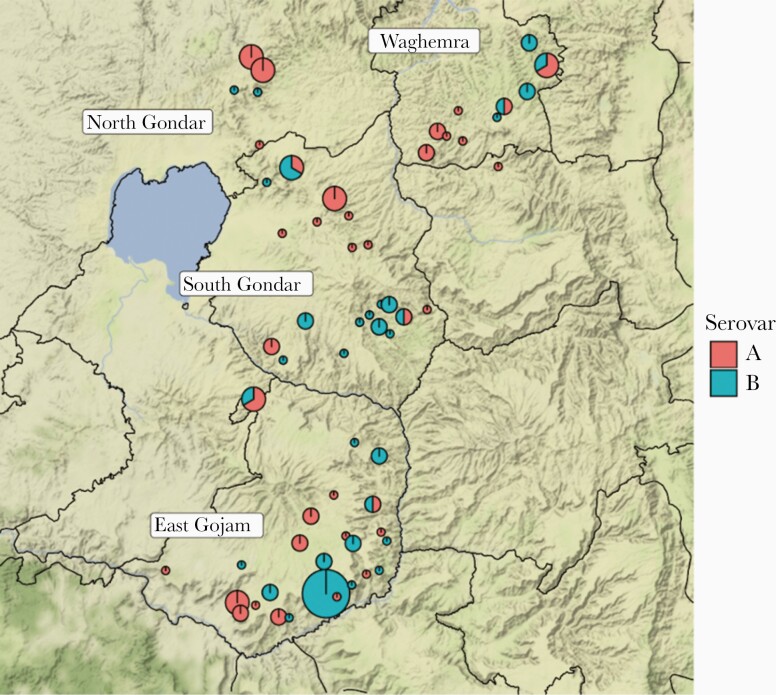
Geographical distribution and similarity of *ompA* serovars. *A*, Four zones in Amhara, Ethiopia were represented in this study. Pie charts represent village-level *Chlamydia trachomatis* prevalence (pie diameter) and presence of *ompA* serovars A (red) and B (blue). Maps were generated using R package ggmap; shape files were obtained from Google Maps.

As *ompA* variation was important in *Ct* phylogeny and heterogeneity in TF profiles, we further investigated the geographical distribution of *ompA* serovars and their relationship to levels of *Ct* infection and TF. SvA and SvB of *ompA* were distributed across all zones (Figure 4). Village-level *Ct* infection, TF, and TI prevalence were not associated with the *ompA* serovar (*P* = .860, .382, and .177, respectively). We identified 9 *ompA* types in this population (Table 2). Six were SvA, defined by 9 nonsynonymous polymorphisms. Three were SvB, defined by 2 nonsynonymous polymorphisms. Four of 9 types were present in all zones (A1, A3, A5, and B3), 4 were exclusive to East Gojam (A2, A4, A6, and B1), and 1 was found in East Gojam and North Gondar (B2) (Supplementary Figure 6). Types A1 (n = 5) and B1 (n = 6) had a nucleotide-predicted amino acid change in the surface-exposed, variable domain (VD) 1, A2 (n = 2) in VD2, and A4 (n = 1) in VD4.

**Table 2.** Description of Nucleotide Polymorphisms and Amino Acid Changes in *ompA* of Amharan *Chlamydia trachomatis* Sequences

*ompA* Type (No.)	Nucleotide Position and Reference Nucleotide (Amino Acid Position and Reference Amino Acid)										
	Serovar A									Serovar B	
	272G	305C	433A	736A	940A	943C	946G	955A	956C	286A	1132G
	(91S)	(102A)	(145T)	(246I)	(314K)	(315P)	(316V)	(319T)	(319T)	(96T)	(378A)
A1 (5)	A (Asp)	…	…	G (Ile)	…	…	…	…	…	…	…
A2 (2)	…	…	G (Ala)	G (Ile)	…	…	…	…	…	…	…
A3 (21)	…	…	…	G (Ile)	…	…	…	…	…	…	…
A4 (1)	…	…	…	…	G (Glu)	G (Ala)	A (Ile)	G (Val)	T (Val)	…	…
A5^a^ (22)	…	…	…	…	…	…	…	…	…	…	…
A6 (1)	…	T (Val)	…	…	…	…	…	…	…	…	…
B1 (6)	…	…	…	…	…	…	…	…	…	G (Ala)	A (Thr)
B2 (6)	…	…	…	…	…	…	…	…	…	…	A (Thr)
B3^a^ (35)	…	…	…	…	…	…	…	…	…	…	…

^a^Reference type per serovar to classify variants.

Most villages (55/61) had only 1 *ompA* type in this study; therefore, we evaluated *ompA* diversity at the district level, using Simpson D and adjusting for number of genomes sampled per district. We used published *Ct* infection, TF, and TI prevalence estimates [6, 28] (Table 3). *Ct* infection and TI prevalence were significantly higher with increasing *ompA* diversity; a similar trend was found for TF prevalence. In a multivariate model, only *Ct* infection prevalence was associated with increasing *ompA* diversity.

**Table 3.** Linear Regression Analysis of Predictors of District-Level *ompA* Diversity

Variable^a^	Univariate			Multivariate		
	β	SE	*P* Value	β	SE	*P* Value
*Ct* infection prevalence	1.134	0.238	.69 × 10^-4^	.402	0.114	.002
TF prevalence	.005	0.004	.159	–.004	0.004	.275
TI prevalence	.033	0.012	.008	.016	0.013	.260

Abbreviations: β, regression coefficient; *Ct*, *Chlamydia trachomatis*; TF, trachomatous inflammation–follicular; TI, trachomatous inflammation–intense; SE, standard error.

^a^District-level prevalence estimates.

## DISCUSSION

This study sequenced *Ct* from ocular samples collected from districts in Amhara, Ethiopia, which had received approximately 5 years of the SAFE strategy, as part of trachoma control efforts. We found that sequences were typical of ocular *Ct*, at both the whole-genome level and in tropism-associated genes, yet phylogenetically distinct from most previously sequenced *Ct* genomes. There was no evidence of macrolide-resistance alleles in this ocular *Ct* population. Greater *ompA* diversity at the district level was associated with increased *Ct* infection prevalence. A continued commitment to the implementation of the SAFE strategy with consideration of enhanced MDA accompanied by further longitudinal investigation is warranted in Amhara.

Almost 900 million doses of azithromycin have been distributed by trachoma control programs since 1999, and in Amhara 15 million doses are administered annually [3]. Mass distribution of azithromycin is likely to become more common as evidence grows of off-target effects such as reducing infectious diseases [21, 29], diarrheal diseases [30], and childhood mortality [20, 31, 32]. There is concern about the impact of these programs on development of antimicrobial resistance [18, 33]. This is particularly true where community-wide treatment with azithromycin has been unable to eliminate trachoma as a public health problem within expected timelines [30, 34]. It has been shown that treating communities with azithromycin can increase nasopharyngeal carriage of macrolide-resistant *Staphylococcus* [35] and *Streptococcus* [36] and alters the fecal microbiome [37, 38], with reports of increased macrolide-resistant *Escherichia coli* [39]. This study, in agreement with previous work [16–18], found no evidence of macrolide resistance in this *Ct* population. While encouraging, it does not rule out macrolide resistance as a potential problem in these communities. Carriage of macrolide-resistant pathogens in the gut and nasopharynx may be impacted by antibiotic treatment. Additionally, presence of additional species of *Chlamydia* [40, 41] and nonchlamydial bacteria [42–45] in the ocular niche have been associated with trachoma; therefore, resistance in other bacteria may be important.

No *Ct* genomes in this study had acquired azithromycin-resistance alleles, but there may be other genomic factors that support *Ct* transmission after treatment. To explore this, we compared Amharan *Ct* genomes with previously sequenced *Ct* to find polymorphism(s) specific to this population that could explain continued transmission. The few SNPs identified as specific to Amhara were dispersed across the genome in known polymorphic genes, rather than being overrepresented in genes related to *Ct* survival. The typical nature of this *Ct* population was supported by phylogenetic clustering with other ocular *Ct* sequences, presence of a nonfunctional tryptophan synthase operon, and tropism-associated polymorphism in *tarP* and the polymorphic membrane proteins. Similar to recent studies from distinct trachoma-endemic communities [12–16, 23], the *Ct* sequences in this population formed 2 closely related subclades within the ocular clade, primarily separated by the *ompA* serovar. Evidence of phylogenetic clustering by country of collection and the similarity to *Ct* sequences collected >50 years prior to this study suggest that diversification in ocular *Ct* is slow and geography related, rather than driven by treatment-derived selection pressure. A surprising finding in this study was that a subgroup of SvB *Ct* from Amhara was most closely related to a historical genome from the United States (Ba/Apache-2) and recently collected genomes from Solomon Islands [23]. It is possible that the origin of these genomes is unique within this population; however, it is more likely that this is further evidence of the slow diversification of *Ct*. In support of this, *ompA* SvB sequences were significantly less diverse than SvA in this study. Furthermore, all major branches of ocular *Ct* phylogeny studied here included samples collected decades apart from geographically disparate sites.

We identified several polymorphic regions associated with village-level TF prevalence. The polymorphisms were mostly frequently found in *ompA*, *pbpB*, and *sufD*, all of which are known to be polymorphic. *OmpA* encodes the major outer membrane protein, which is the primary target of host immune responses and is believed to function as an adhesin and/or porin [46]. The functions of *pbpB* and *sufD* in *Ct* are unknown; bacterial homologues of these genes function in peptidoglycan synthesis and response to oxidative stress, respectively. It is plausible that genes hypothesized to be involved in immune evasion and response to stress could impact *Ct* survival and response to treatment.

We found approximately equal representation of SvA and SvB in this study. Both serovars were present in all districts and were not associated with village-level *Ct* infection, TF, or TI prevalence. However, *Ct* infection prevalence was increased in districts with greater *ompA* diversity. Our data agree with a Nepalese study that found increased *ompA* diversity in villages to be associated with higher trachoma prevalence [47]. In contrast, a more recent study from Ethiopia found no association between *ompA* diversity and *Ct* infection levels [48]. It is known that immunity to *Ct* is serovar specific [49, 50]; therefore, it is plausible that in villages with multiple serovars in circulation, individuals are more likely to be exposed to a serovar they do not have protective immunity against. Presence of 1 or more *ompA* variants should not impact treatment success; however, it is possible that higher levels of *Ct* infection pretreatment, driven by presence of multiple serovars, could increase the likelihood of low-level transmission persisting after treatment.

A potential limitation of this study was bias toward samples with higher *Ct* load. It is possible that relationships between *ompA* variation and *Ct* infection prevalence might have been different if lower load infections were included, particularly at the village level, as the majority (34/61) were represented by 1 sequence. We have also not sequenced material from Abbott m2000 specimens previously, so it is possible that long-term storage in this format and multiple freeze-thaw cycles may have impacted DNA quality or quantity. However, obtaining high-quality genomes from all sequenced samples, with as low as 500 *Ct* genomes input, suggests that quality was not an issue. Additionally, our sample size was restricted by both *Ct* load and the cost of sequencing. This is the largest collection of ocular *Ct* genomes from a single geographical population, but it is still possible that we may have missed some smaller effects in the genome-wide analyses due to limited statistical power. Last, while study villages were randomly selected using standard programmatic methods and subsampling was matched for zone of collection, the conclusions of this study may have been different if we had been able to sequence a larger and more geographically diverse population of *Ct.*

Despite approximately 5 years of azithromycin MDA, we found no evidence for *Ct* genomic variation contributing to continued transmission of *Ct*, adding to evidence that azithromycin MDA does not drive acquisition of macrolide-resistance alleles in *Ct*. This study demonstrates feasibility of WGS of low-load, residual material and highlights the added value of collecting ocular swabs as part of trachoma surveys. Collection and long-term storage of these samples has helped alleviate concerns of azithromycin resistance in Amharan *Ct*, while offering important insights into the relationship between *ompA* variation and *Ct* infection levels. Future longitudinal investigations will be needed to understand what impact *ompA* diversity may have on treatment success in Amhara and other trachoma-endemic regions.

## Supplementary Data

Supplementary materials are available at *The Journal of Infectious Diseases* online. Consisting of data provided by the authors to benefit the reader, the posted materials are not copyedited and are the sole responsibility of the authors, so questions or comments should be addressed to the corresponding author.

jiaa615_suppl_Supplementary_Figure_S1

jiaa615_suppl_Supplementary_Figure_S2

jiaa615_suppl_Supplementary_Figure_S3

jiaa615_suppl_Supplementary_Figure_S4

jiaa615_suppl_Supplementary_Figure_S5

jiaa615_suppl_Supplementary_Figure_S6

jiaa615_suppl_Supplementary_File

## Notes

***Author contributions.*** H. P., R. L. B., E. K. C., M. J. H., and S. D. N. contributed to study design. H. P., C. A. W., A. C., E. S., M. Z., Z. T., E. K. C., and S. D. N. contributed to data collection. H. P., A. W. N., E. K. C., M. J. H., and S. D. N. contributed to data analysis. All authors interpreted the findings, contributed to writing the manuscript, and approved the final version.

***Acknowledgments.*** The authors acknowledge the study participants and field team in Amhara, Ethiopia. The authors also acknowledge the infrastructure support provided by the University College London/University College London Hospitals Biomedical Research Centre–funded Pathogen Genomics Unit. The authors thank Abbott for donation of the m2000 RealTime molecular diagnostics system and consumables. All sequence data are available from the European Bioinformatics Institute archives (PRJEB38668).

***Financial support.*** This work received financial support from the Coalition for Operational Research on Neglected Tropical Diseases, which is funded at the Task Force for Global Health by the Bill & Melinda Gates Foundation, the United Kingdom Department for International Development, and the United States Agency for International Development. Additional financial support was received from the International Trachoma Initiative. H. P. and M. H. were funded by EU Horizon 2020 (grant number 733373).

***Potential conflicts of interest.*** All authors: No reported conflicts of interest.
