# Supplementary figures and images for: Genomics of Ocular Chlamydia trachomatis After 5 Years of SAFE Interventions for Trachoma in Amhara, Ethiopia
Source: J Infect Dis. 2020 Oct 9;225(6):994–1004. doi: 10.1093/infdis/jiaa615 (PMC8922003; doi:10.1093/infdis/jiaa615)

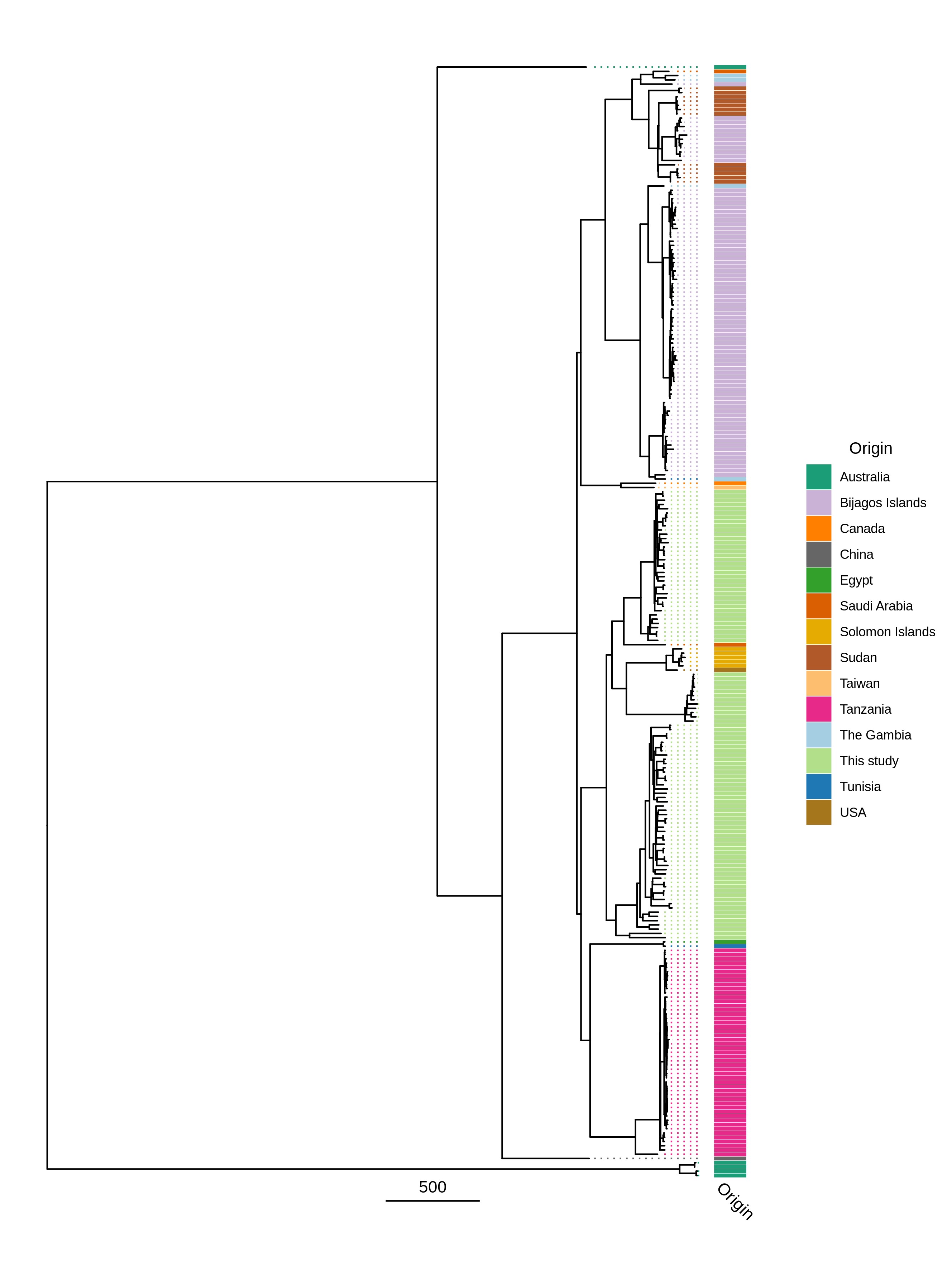

Supplement: jiaa615_suppl_Supplementary_Figure_S1 [file jiaa615_suppl_supplementary_figure_s1.jpeg]

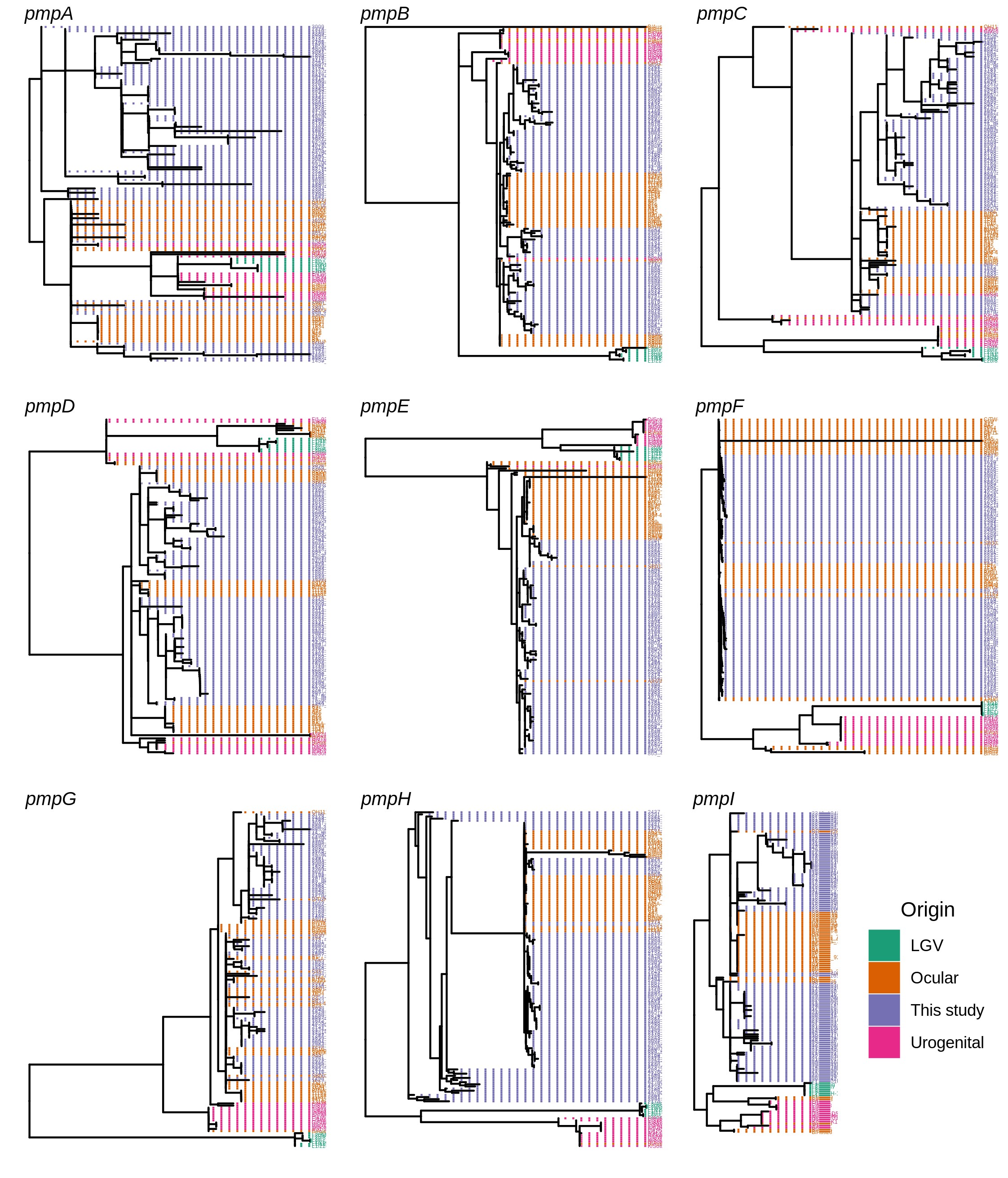

Supplement: jiaa615_suppl_Supplementary_Figure_S2 [file jiaa615_suppl_supplementary_figure_s2.jpeg]

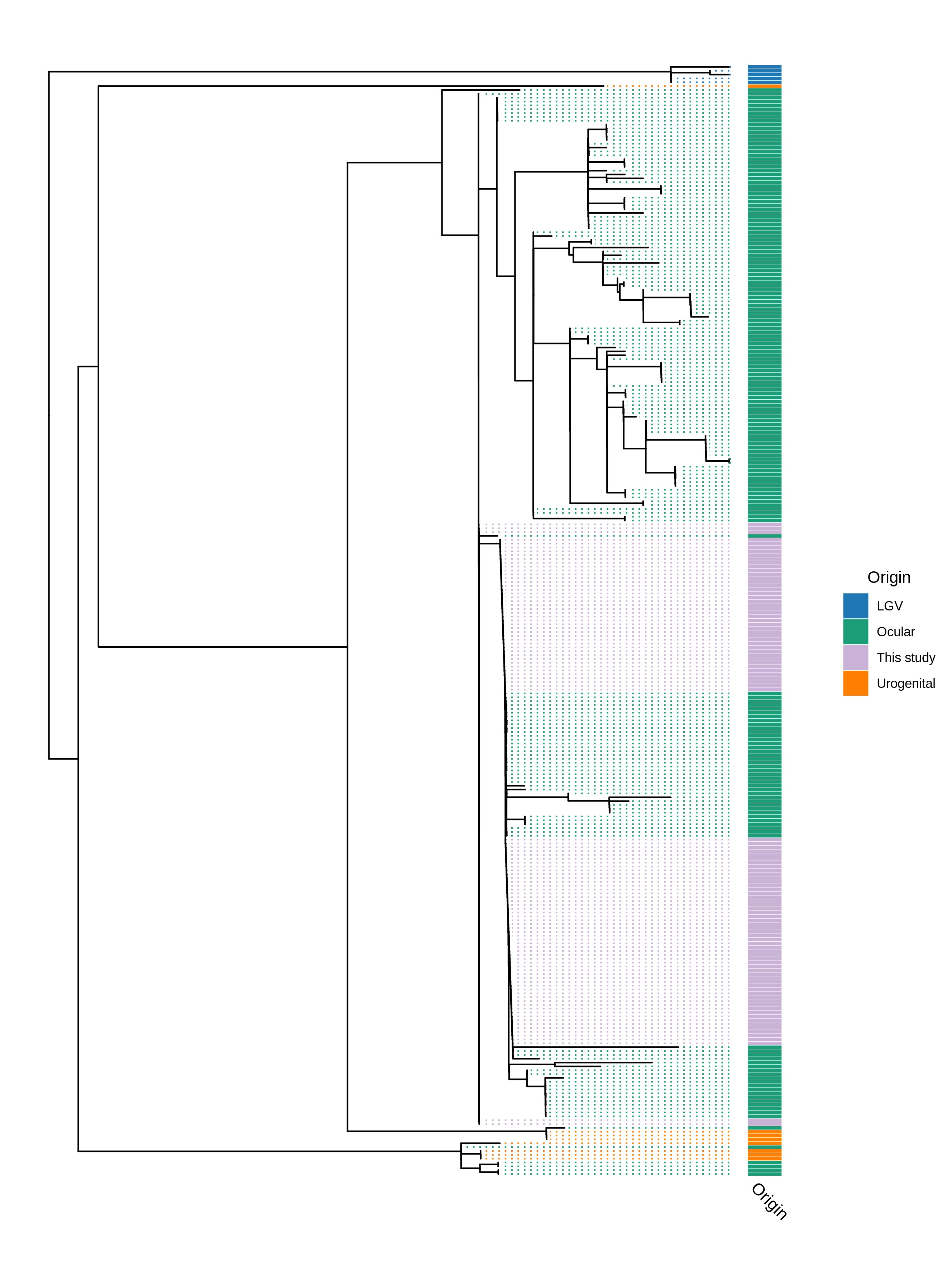

Supplement: jiaa615_suppl_Supplementary_Figure_S3 [file jiaa615_suppl_supplementary_figure_s3.jpeg]

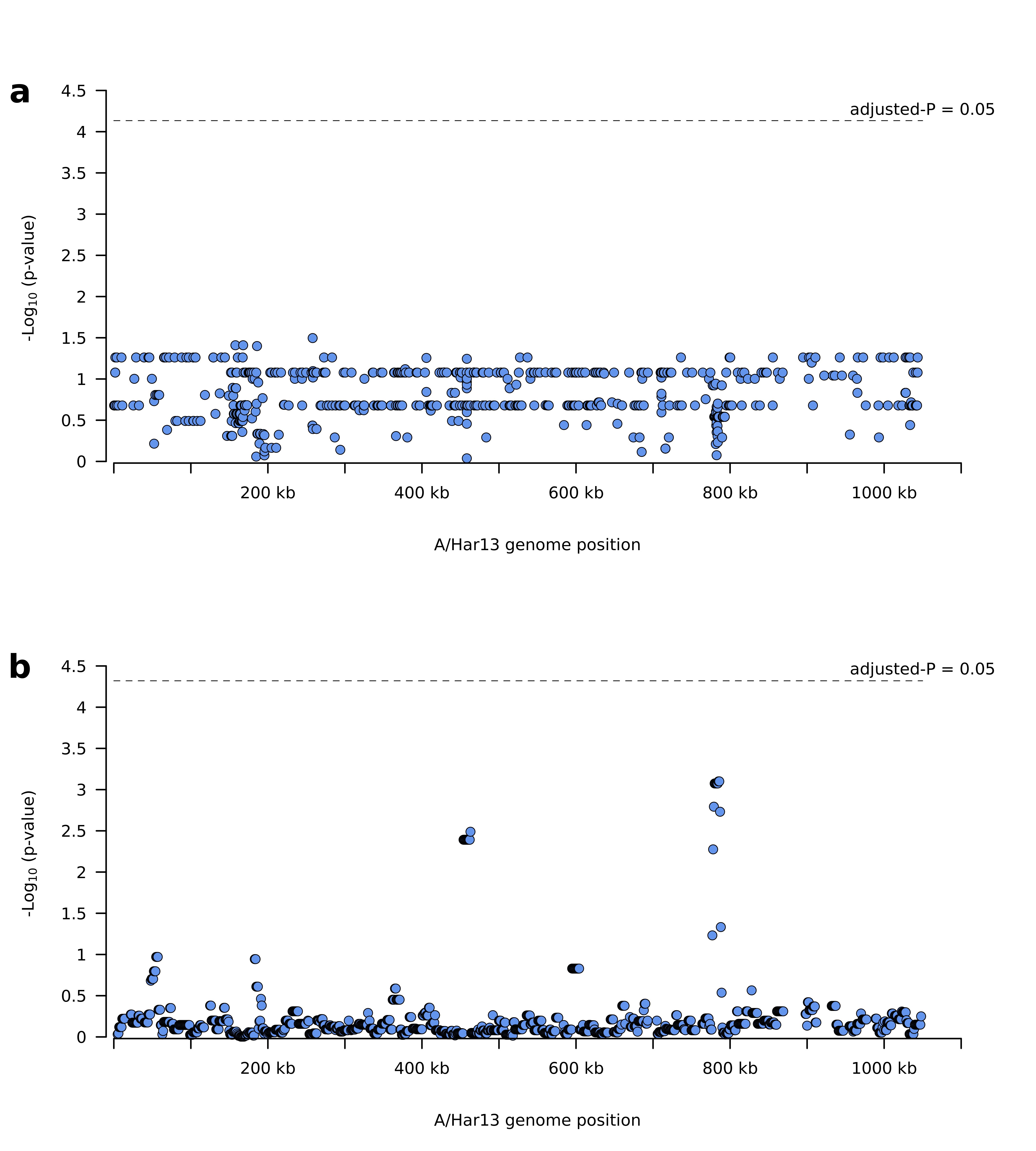

Supplement: jiaa615_suppl_Supplementary_Figure_S4 [file jiaa615_suppl_supplementary_figure_s4.jpeg]

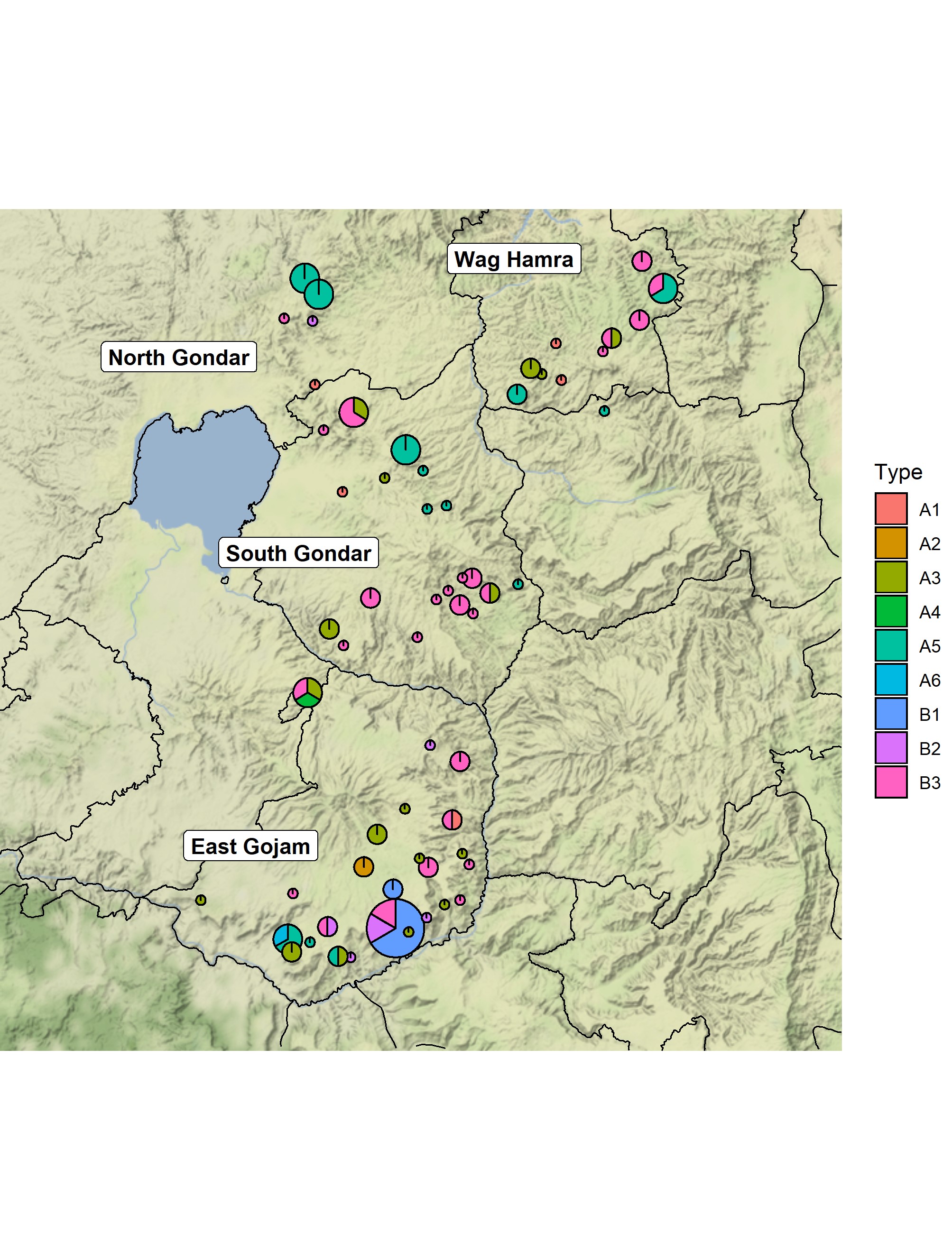

Supplement: jiaa615_suppl_Supplementary_Figure_S5 [file jiaa615_suppl_supplementary_figure_s5.jpeg]

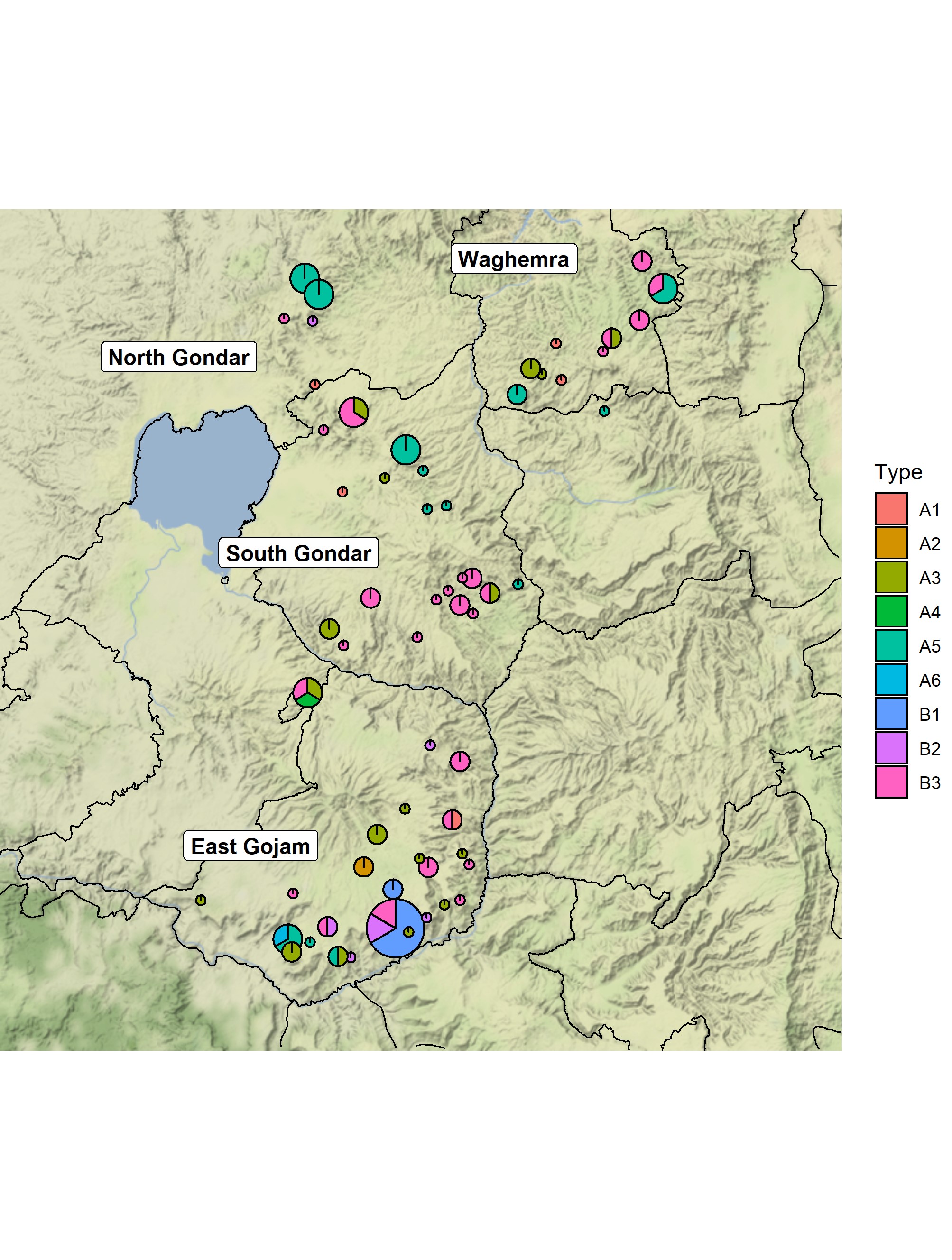

Supplement: jiaa615_suppl_Supplementary_Figure_S6 [file jiaa615_suppl_supplementary_figure_s6.jpeg]
