# Supplementary material for: Genomics of Ocular Chlamydia trachomatis After 5 Years of SAFE Interventions for Trachoma in Amhara, Ethiopia
Source: J Infect Dis. 2020 Oct 9;225(6):994–1004. doi: 10.1093/infdis/jiaa615 (PMC8922003; doi:10.1093/infdis/jiaa615)
Supplement: jiaa615_suppl_Supplementary_File [file jiaa615_suppl_supplementary_file.docx]

***Additional File 1.***

Maximum likelihood reconstruction of *ompA* phylogeny of ocular Chlamydia trachomatis (Ct) sequences from Amhara, Ethiopia. Phylogeny of *ompA* from 99 *Ct* sequences from Amhara and 183 *Ct* clinical and reference strains. Amharan *Ct* sequences were mapped to *Ct* A/HAR-13 using Bowtie2. SNPs were called using SAMtools/BCFtools. Phylogenies were computed with RAxML from a variable sites alignment using a GTR + gamma model and are midpoint rooted. The scale-bar indicates pairwise distance. *Ct* sequences are coloured by country of origin of the sample (“Origin”).

***Additional File 2.***

Maximum likelihood reconstruction of phylogeny of the polymorphic membrane proteins (pmps) of ocular *Chlamydia trachomatis* (*Ct*) sequences from Amhara, Ethiopia. Phylogeny of *ompA* from 99 *Ct* sequences from Amhara and 183 *Ct* clinical and reference strains. Amharan *Ct* sequences were mapped to *Ct* A/HAR-13 using Bowtie2. SNPs were called using SAMtools/BCFtools. Phylogenies were computed with RAxML from a variable sites alignment using a GTR + gamma model and are midpoint rooted. *Ct* sequences are coloured by country of origin of the sample (“Origin”).

***Additional File 3.***

Maximum likelihood reconstruction of plasmid phylogeny of ocular *Chlamydia trachomatis* (*Ct*) sequences from Amhara, Ethiopia. Phylogeny of *ompA* from 99 *Ct* sequences from Amhara and 183 *Ct* clinical and reference strains. Ethiopian *Ct* sequences were mapped to *Ct* A/HAR-13 using Bowtie2. SNPs were called using SAMtools/BCFtools. Phylogenies were computed with RAxML from a variable sites alignment using a GTR + gamma model and are midpoint rooted. *Ct* sequences are coloured by country of origin of the sample (“Origin”).

***Additional File 4.***

Polymorphisms on the *Chlamydia trachomatis* (*Ct*) genome associated with village-level *Ct* infection prevalence. a) No single nucleotide polymorphisms were significantly associated with village-level *Ct* infection prevalence. b) No polymorphic regions were significantly associated with village-level *Ct* infection prevalence.

***Additional File 5.***

Polymorphisms on the *Chlamydia trachomatis* (*Ct*) genome associated with village-level TI prevalence. a) No single nucleotide polymorphisms were significantly associated with village-level TI prevalence. b) No polymorphic regions were significantly associated with village-level TI prevalence.

***Additional File 6.***

Geographical distribution of *ompA* types. Four zones in Amhara, Ethiopia were represented in this study. Pie charts represent village-level *Ct* prevalence (pie diameter) and presence of *ompA* types. Maps were generated using R package ggmap, shape files were obtained from Google Maps.
